# Grading of Hydronephrosis: An Ongoing Challenge

**DOI:** 10.3389/fped.2020.00458

**Published:** 2020-08-27

**Authors:** Abdurrahman Onen

**Affiliations:** Section of Pediatric Urology, Department of Pediatric Surgery, Faculty of Medicine, Dicle University, Diyarbakir, Turkey

**Keywords:** children, hydronephrosis, ureteropelvic junction obstruction, grading, treatment, surgery

## Abstract

The crucial point for prompt diagnostics, ideal therapeutic approach, and follow-up of hydronephrosis associated with UPJ anomalies in children is the severity of hydronephrosis. Such many hydronephrosis grading systems as AP diameter, SFU, radiology, UTD, and Onen have been developed to evaluate hydronephrosis severity in infants. Unfortunately, it is still an ongoing challenge and there is no consensus between different disciplines. AP diameter is a very dynamic parameter and is affected by many factors (hydration, bladder filling, position, respiration). More importantly, its measurement is very variable and misleading due to different renal pelvic configurations. The radiology grading system has the same grades 1, 2, and 3 as the SFU grading system with addition of the AP diameter for the first 3 grades. This grading system divides parenchymal loss into two different grades. Grade 4 represents mild parenchymal loss while grade 5 suggests severe parenchymal loss. However, it is operator dependent, is not decisive, and does not differentiate grades 4 and 5 clearly. All grades of SFU are very variable between operators and clinicians. UTD classification aims to put all significant abnormal urinary findings together including the kidney, ureter, and bladder and thus determines the risk level for infants with any urinary disease. Different renal deterioration risks occur depending on the mechanism of hydronephrosis. Therefore, SFU and UTD classification may result in significant confusion and misleading in determining the severity of hydronephrosis. SFU-4 and UTD-P3 represent a considerable range of severity of hydronephrosis. Both represent minimal thinning of the medullary parenchyma and severe thinning of the cortical parenchyma (cyst-like hydronephrotic kidneys) at the same grade. The wide definition of SFU-4 and UTD-P3 fails to indicate accurately the severity of hydronephrosis and thus significantly misleads from a prompt treatment. They do not suggest who need surgical treatment and who can safely be followed non-operatively. The anatomy and physiology of the 4 suborgans of the kidney (renal pelvis, calices, medulla, and cortex) are completely different from each other. Therefore, each part of the kidney affect and behave differently as a response to UPJ-type hydronephrosis (UPJHN) depending on the severity of hydronephrosis. The upgraded Onen hydronephrosis grading system has been developed based on this basic evidence both for prenatal and post-natal periods. The Onen grading system determines specific detailed findings of significant renal damage, which clearly show and suggest who can safely be followed conservatively from who will need surgical intervention for UPJHN. Neither AP diameter nor radiology, SFU, or UTD classification is the gold standard in determining the severity of hydronephrosis. All these grading systems are based on subjective parameters and are affected by many factors. They do not determine the exact severity of UPJHN and thus cause permanent renal damage due to a delay in surgical decision in some infants while they may cause an unnecessary surgery in others. The Onen grading system has resolved all disadvantages of other grading systems and promises a safer follow-up and a prompt treatment for UPJHN. It is an accurate and easily reproducible grading that has high sensitivity and specificity.

## Introduction

Urinary ultrasound (US) is the best we have for the diagnosis and follow-up of both prenatal and post-natal hydronephrosis as a similar modality (1–11). It is non-invasive, easily available, fast, and low-cost; can be performed directly in bedside manner; and does not involve radiation. It shows the size of kidneys, thickness, and appearance of parenchyma (echogenicity, corticomedullary differentiation, cortical cysts), severity of hydronephrosis, ureteral dilation, and bladder anatomy (1, 2, 4–6, 9–11).

Ultrasound not only gives anatomic details but also gives some functional clues about the urinary system. It, therefore, provides excellent diagnostic accuracy. There are two important benefits of ultrasound: It determines the severity of hydronephrosis promptly and the time and necessity of other diagnostics (1, 3–6, 8, 10–12).

We need to determine specific criteria and risky findings suggestive of renal damage, which help clinicians to decide a prompt therapeutic approach. In this review, we will outline the most recent criteria to accurately determine the severity of hydronephrosis and thus predict who may develop renal damage and need intervention compared with who can safely be followed conservatively.

## Anatomo-Physio-Pathology of Ureteropelvic Junction Type Hydronephrosis (Upjhn)

The kidney has 2 main parts: The most important part is the renal parenchyma which does function and produce urine. The other is the pelvicaliceal system which collects and sends urine into the ureter. The renal parenchyma has two suborgans: medulla and cortex. The collecting system has two suborgans: renal pelvis and calices.

Two factors affect the kidney in infants with UPJHN: the compliance of renal pelvis and the degree of stenosis at UPJ. First, hydronephrosis develops as a protecting anatomic response. If the stenosis is severe and persists for a long period, then renal damage occurs as a functional response (1, 4, 11).

The anatomy and physiology of renal suborgans (renal pelvis, calices, medulla, and cortex) are completely different from each other. Therefore, each part affects and behaves differently as a response to UPJHN depending on the severity of hydronephrosis.

- *Renal pelvis*: The compliance of renal pelvis is very high in infants. It is particularly true for those who have extrarenal pelvic configuration due to their high expandability. The renal pelvis enlarges significantly to protect the renal parenchyma even in mild increase at renal pelvic pressure. Therefore, the risk of renal parenchymal damage is low and takes time in such infants comparatively. However, the risk of renal damage is high in those who have intra-renal pelvic configuration due to their low compliance.
- *Calices*: The expandability of calices is lower than that of the renal pelvis. Their compliance is low comparatively. Therefore, the dilation of calices means a greater degree (risk) of hydronephrosis compared to renal pelvic dilation alone. On the other hand, the calices enlarge to protect the renal parenchyma.
- *Medulla:* Its structure is somewhat similar to that of the lung. This part of the renal parenchyma is more expandable and compressed rapidly compared to the renal cortex. Depending on the degree of UPJ stenosis and time interval, the medulla becomes shorter and loses its pyramid form. The lower limit of the normal renal parenchymal thickness is 7.5 mm at the neonatal period, 8 mm at 1 year of age, and 10 mm at 2 years of age (10).
- *Cortex:* It is the most important functional part of the kidney. The normal thickness of the cortex is > 3 mm in infants. Its structure is somewhat similar to that of the liver, which is a relatively hard solid organ. Therefore, its compression or thinning means there is a significant risk of renal damage. In such cases, corticomedullary differentiation is lost and the thickness of the cortex decreases. It is an objective parameter because, opposite of the pelvicaliceal system, it is not affected from hydration, bladder filling, position, and respiration. The measurement points are not controversial and are not operator dependent. The renal parenchyma is measured at the thinnest point of the parenchyma on the longitudinal section of the kidney (1, 4, 5, 7, 10). It does not have intraobserver or interobserver variation (1, 10, 11, 13). Long-lasting cortical thinning is associated with low renal function and decrease in the number of nephrons (1, 4, 5, 11, 14). Therefore, the compressed and thinned cortex is suggestive of renal damage. The loss of more than half of the cortex (cortex thickness <1.5 mm) is mostly associated with renal atrophy and irreversible renal damage.

The quality of the renal parenchyma which includes the *thickness* and *appearance* of the parenchyma is the most important and objective parameter to determine kidney exposure and thus the severity of hydronephrosis.

- *Thickness of the renal parenchyma*: Severe cortical damage (dilation, epithelial apoptosis, and atrophy of the renal tubules, and inflammation and fibrosis of the glomerulus) and decrease in glomerular filtration and renal function occur in infants, developing parenchymal loss due to severe UPJHN (14). The incidence of permanent functional loss is high (8–16%) while histopathological changes do not improve even after a successful pyeloplasty in infants with severe parenchymal loss which delayed surgery (1, 4, 5, 11, 15, 16). Loss of the renal cortex and reduced renal size are the result of tubular atrophy and correlate with chronic irreversible renal disease (15). The number of nephrons decreases, renal maturation is affected, and renal failure occurs in such cases (17).
- *Appearance of the renal parenchyma*: Hyperechogene parenchyme, cystic degeneration in the cortex, and loss of corticomedullary differentiation on ultrasound are findings suggesting significant renal damage, which are compatible with decrease in renal function on scintigraphy (1, 11). Cortical echogenicity is a parameter that correlates well with tubular atrophy and interstitial inflammation (15).

Another important parameter is the longitudinal length of both normal and hydronephrotic kidneys. The compensatory hypertrophy of the contralateral kidney (length > 20% of normal) means affected kidney worsening even if Onen-3 hydronephrosis is stable. The longitudinal length of the affected kidney should be higher than the normal value, depending on the severity of hydronephrosis. If the affected kidney length stays in the normal range despite severe hydronephrosis, it means the affected kidney undergoes atrophy.

## Hydronephrosis Grading Systems

### Anterior–Posterior (AP) Diameter of Renal Pelvis (APDRP)

The measurement of the AP diameter of the renal pelvis is not standardized between different disciplines, and there is a consensus only in 64% of physicians (10, 18). Unfortunately, it is significantly operator dependent. Some sonographers measure the AP diameter at the largest point of the renal pelvis while others measure it at vertical plan. However, the APDRP is mostly measured at the parenchymal edge (hilus) during the transverse section of the kidney.

The renal pelvis and AP diameter is very dynamic; its measurement changes significantly depending on hydration, bladder filling, position (supine or prone), and respiration (1, 10, 12, 18, 19).

More importantly, its measurement is very variable and misleading due to different renal pelvic configurations. Hydronephrosis may be moderate even if the AP diameter is high in infants with extrarenal pelvic configuration. On the other hand, hydronephrosis may be very severe with significant parenchymal thinning even if the AP diameter is low in infants with intrarenal pelvic configuration. Therefore, if the quality of parenchyma which is the most important factor in determining the degree of hydronephrosis is omitted and the AP diameter itself is accepted as the only finding for severity of hydronephrosis, then some infants may undergo an unnecessary surgery while some may result in permanent renal damage due to a delay for prompt surgery.

Disadvantages/limitations of APDRP:

- The rate of operator differences is very high
- AP diameter is low in dehydrated infants
- AP diameter is low in the empty bladder
- AP diameter is low in the expirium phase
- AP diameter is less ideally measured in supine position
- AP diameter (even low) is very risky in the presence of intrarenal pelvic configuration.

### SFU Grading System

**Figure 1:**
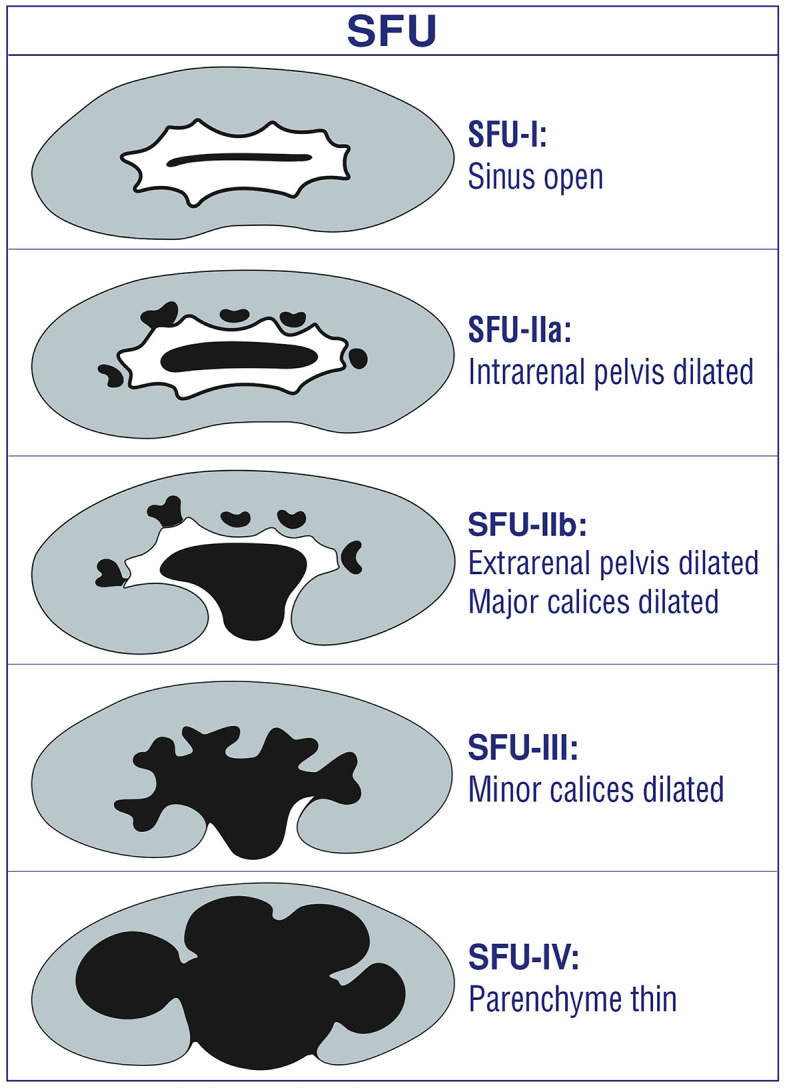
SFU hydronephrosis grading system.

This grading system has been developed in 1993 (9) (Figure 1). It is quantitative and subjective. All grades of SFU are very variable between operators and clinicians (1, 4–6, 10, 11, 20–22). Therefore, it is not popular between disciplines other than pediatric urologists (1, 4, 5, 7, 10, 11, 19–21, 23–25).

Disadvantages/limitations of SFU

- *SFU-1 and SFU-2a:* Both indicate different degrees of renal pelvic dilation. Therefore, it is confusing and very difficult to differentiate each other (1, 2, 4). Moreover, follow-up, treatment, and prognosis of these two degrees are similar; all of them resolve spontaneously without renal damage (1, 2, 4, 5, 20).
- *SFU-2b and SFU-3*: Both represent different degrees of calyceal dilation. It is very operator dependent in differentiating the dilation of peripheral (minor) calices from those of central (major) calices due to a high discrepancy within and between raters for interpretation of the two types of calyceal dilation (26, 27). Therefore, it is subjective and confusing and it is very difficult to differentiate each other (1, 4).
- *SFU-3:* Although it represents only calyceal dilation, the pictures used for SFU-3 in the original article clearly show severe medullary thinning. This causes significant confusion among clinicians and radiologists.
- *SFU-4:* It represents minimal thinning of the medullary parenchyma (e.g., 6 mm) and severe thinning of the cortical parenchyma (e.g., 2 mm) and cyst-like hydronephrotic kidneys at the same grade (2). The wide definition of SFU-4 fails to demonstrate accurately the severity of hydronephrosis and thus significant misleads from a prompt treatment. It does not suggest who need surgery and who can safely be followed non-operatively. The first example (medulla thin) can safely be followed non-operatively while the second (cortex thin) clearly need surgery. This wide definition makes prognosis difficult to predict in UPJHN cases (1, 4–6, 8, 11, 28).

### Radiology Grading System

**Figure 2:**
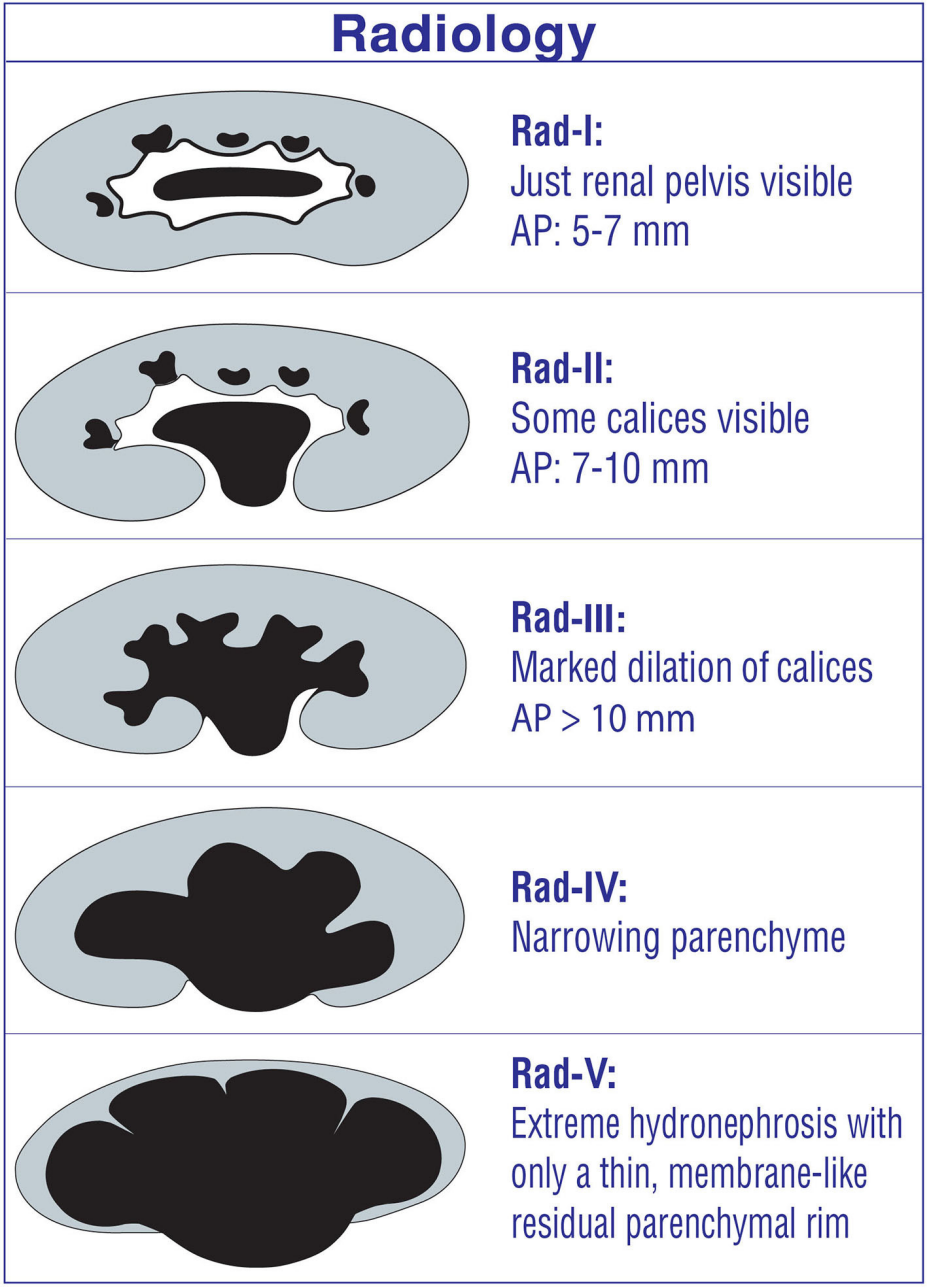
Radiology hydronephrosis grading system.

The radiology grading system has partially been modified from SFU for post-natal use (7, 9) (Figure 2). It has the same grades 1, 2, and 3 as the SFU grading system (8, 14). In addition, it includes AP diameter for the grades 1, 2, and 3. This grading system divides parenchymal loss into two different grades, suggesting the importance of the renal parenchyma to determine the severity of hydronephrosis which has a somewhat similar idea as in the Onen grading system (1, 4, 7). Grade 4 hydronephrosis represents mild parenchymal loss; grade 5, severe parenchymal loss.

Disadvantages/limitations of the radiology grading system

- *Radiology grades 1 and 2 (SFU-1 and SFU-2a):* Both indicate different degrees of renal pelvic dilation. Therefore, it is confusing and very difficult to differentiate each other (1, 2, 4). Moreover, follow-up, treatment, and prognosis of these two degree are similar; all of them resolve spontaneously without renal damage (1, 2, 4, 5, 20).
- *The usage of the AP diameter:* It makes this grading system even more confusing, because SFU grades and AP diameter are not parallel for many patients depending on different renal pelvic configurations. In addition, the AP diameter is affected significantly by many factors as previously described in this review (1, 10, 12, 18, 19).
- *Radiology grades 4 and 5:* Grade 4 represents mild parenchymal loss, while grade 5 represents severe parenchymal loss. It is completely operator dependent, is not decisive, and does not differentiate grades 4 and 5 clearly. Therefore, between- and intra-rater reliability is low.

### UTD Classification

**Figure 3:**
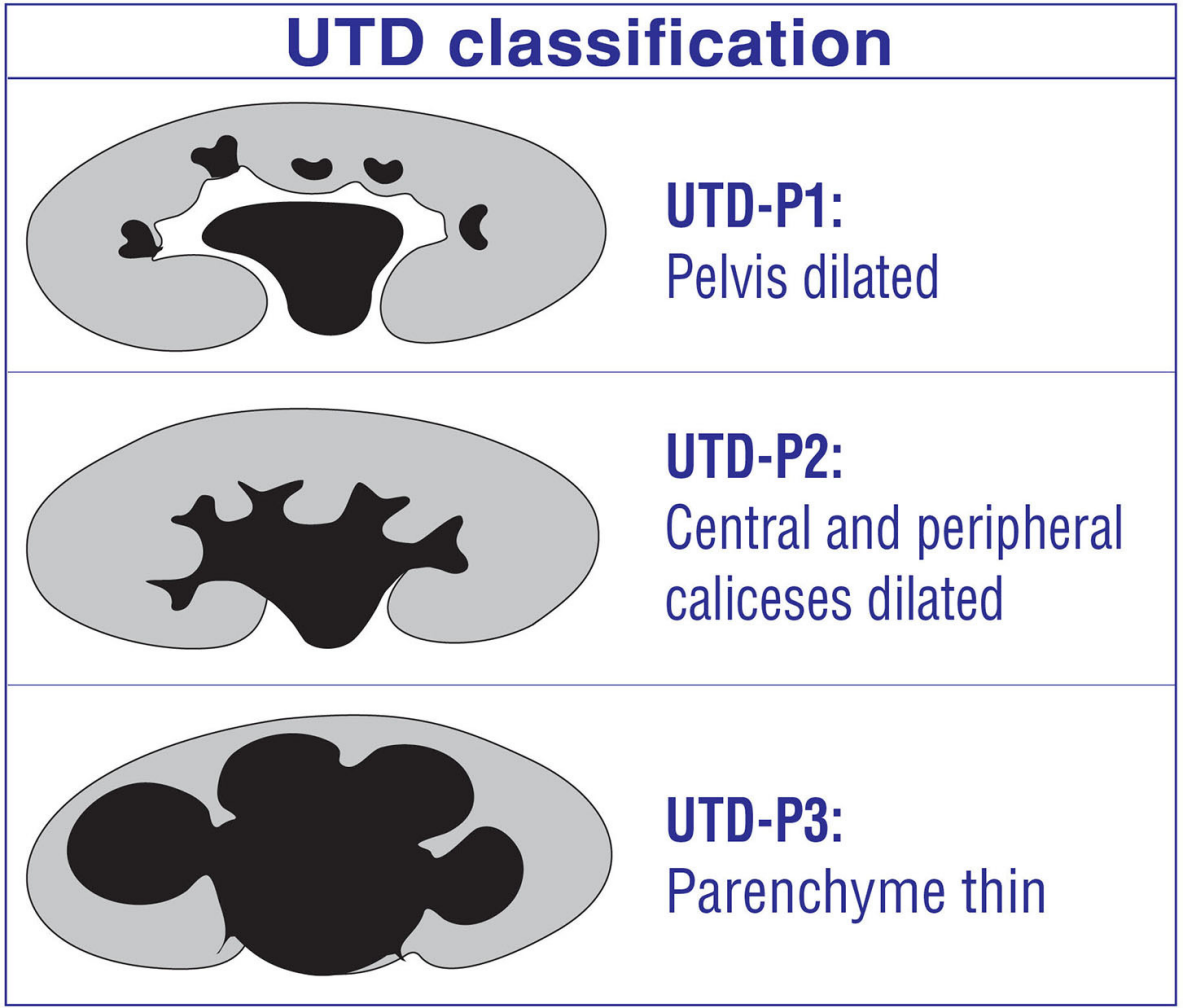
UTD classification for post-natal hydronephrosis.

UTD has been created retrospectively based on reviewing, combining, and summarizing the current literature (2) (Figure 3). It, therefore, is not an evidence-based grading system. Actually, it most likely has been modified from SFU and Onen grading systems (4, 9). It aims to put all significant abnormal urinary findings together including the kidney, ureter, and bladder and thus determines the risk level for a hydronephrotic infant with any kind urinary diseases. It includes such parameters as AP diameter of renal pelvis, central and peripheral calyceal dilation, renal parenchyma, ureteral abnormalities, and bladder abnormalities (2). All these findings are very important by themselves. However, the natural history, diagnosis, follow-up, treatment, and prognosis of urinary diseases are significantly different from each other depending on the etiopathology of hydronephrosis.

This classification suggests the general term “urinary tract dilation” to indicate ultrasound findings that include all ureteral and kidney dilations (2). It is clear that UPJ-type hydronephrosis, UVJ-type hydroureteronephrosis, vesicoureteral reflux, bladder pathologies (ureterocele, diverticula, etc.), and posterior urethral valve cause hydronephrosis in very different ways. They may cause different levels and types of renal damage and prognosis (1, 4, 5). For example, Onen-3 (medulla thin) hydronephrosis due to UPJHN can be followed non-operatively while an infant with the same degree of hydronephrosis due to grade 5 reflux has a much higher risk of UTI, renal scar, and surgical need (1). Different renal deterioration risks occur depending on the mechanism of hydronephrosis. Therefore, UTD classification may result in significant confusion and mislead in determining the severity of hydronephrosis (1).

Disadvantages/limitations of UTD classification

- *Central and peripheral calices:* It is very operator dependent to differentiate the dilation of peripheral (minor) calices from those of central (major) calices due to a high discrepancy within and between raters for interpretation of the two types of calyceal dilation (26, 27). Therefore, it is subjective and confusing and is very difficult to differentiate each other (1, 4).
- *UTD-P3:* Like SFU, it represents minimal thinning of the medullary parenchyma (e.g., 6 mm) and severe thinning of the cortical parenchyma (e.g., 2 mm) and cyst-like hydronephrotic kidneys at the same grade (2). The wide definition of UTD-P3 fails to demonstrate accurately the severity of hydronephrosis and thus significant misleads from prompt treatment. It does not suggest who need surgical treatment and who can safely be followed non-operatively. The first example (medulla thin) can safely be followed non-operatively while the second (cortex thin) clearly need surgery. This wide definition makes prognosis difficult to predict in UPJHN cases (1, 4–6, 8, 11, 28).

### Onen Grading System

**Figure 4:**
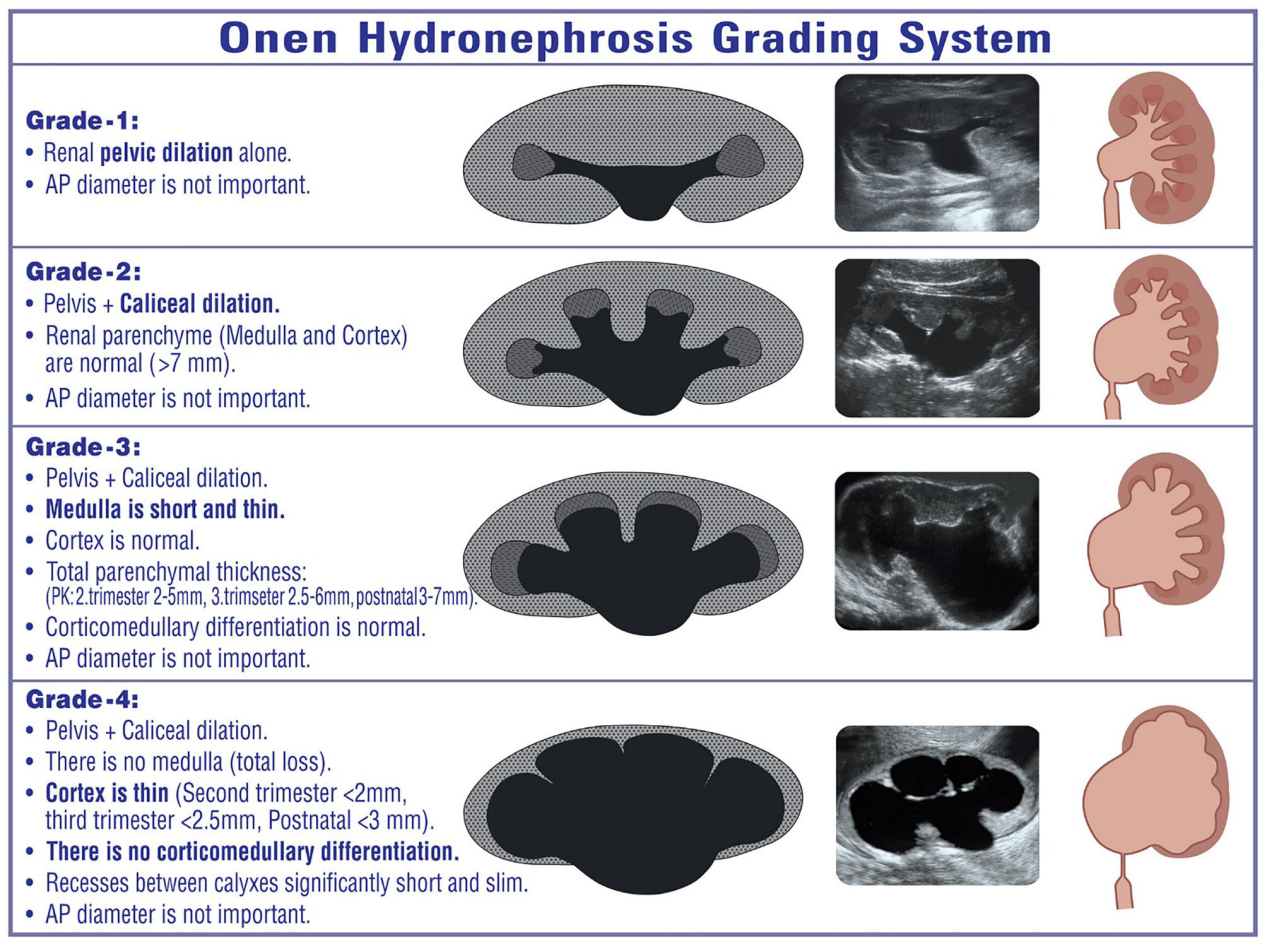
Onen hydronephrosis grading system for both prenatal and post-natal UPJHN.

This grading system has been developed for both prenatal and post-natal UPJHN (Figure 4). It is appropriate and applicable for both fetus and children, which standardize the language of the sonographers, clinicians, method of evaluation, and measurement of kidneys. The Onen grading system is terminologically simple and clear. Therefore, all disciplines including radiology, perinatology, pediatric nephrology, and pediatric urology can easily use not only for clinical practice but also for future researches.

The Onen grading system has evidence-based standardized objectives and reproducible parameters (4). It includes two categories of kidney findings. The first is dilation of the pelvicalyceal system; the second which is the most important category is the quality of the renal parenchyma (thickness and appearance) (1). This grading system divides thinning of the renal parenchyma into two grades: medullary thinning and cortical thinning. In addition, the appearance of the parenchyma (echogenicity, cortical cysts, corticomedullary differentiation) which is suggestive of renal damage is also taken into account in this grading system.

It was proposed on the basis of a well-known tight association between the severity of hydronephrosis and prognosis; renal deterioration may occur in severe hydronephrosis not timely and promptly treated (1, 4–6, 8, 11, 23, 29, 30). This grading system is beneficial in determining the possible risk of renal damage, surgical necessity, and prognosis in infants with UPJHN. Therefore, such cases can safely be followed based on this grading system. Because it determines clearly those infants who can be followed with ultrasound alone, who need renal scan, and who require surgery.

Our treatment and follow-up protocol for UPJHN based on the Onen grading system

**Figure 5:**
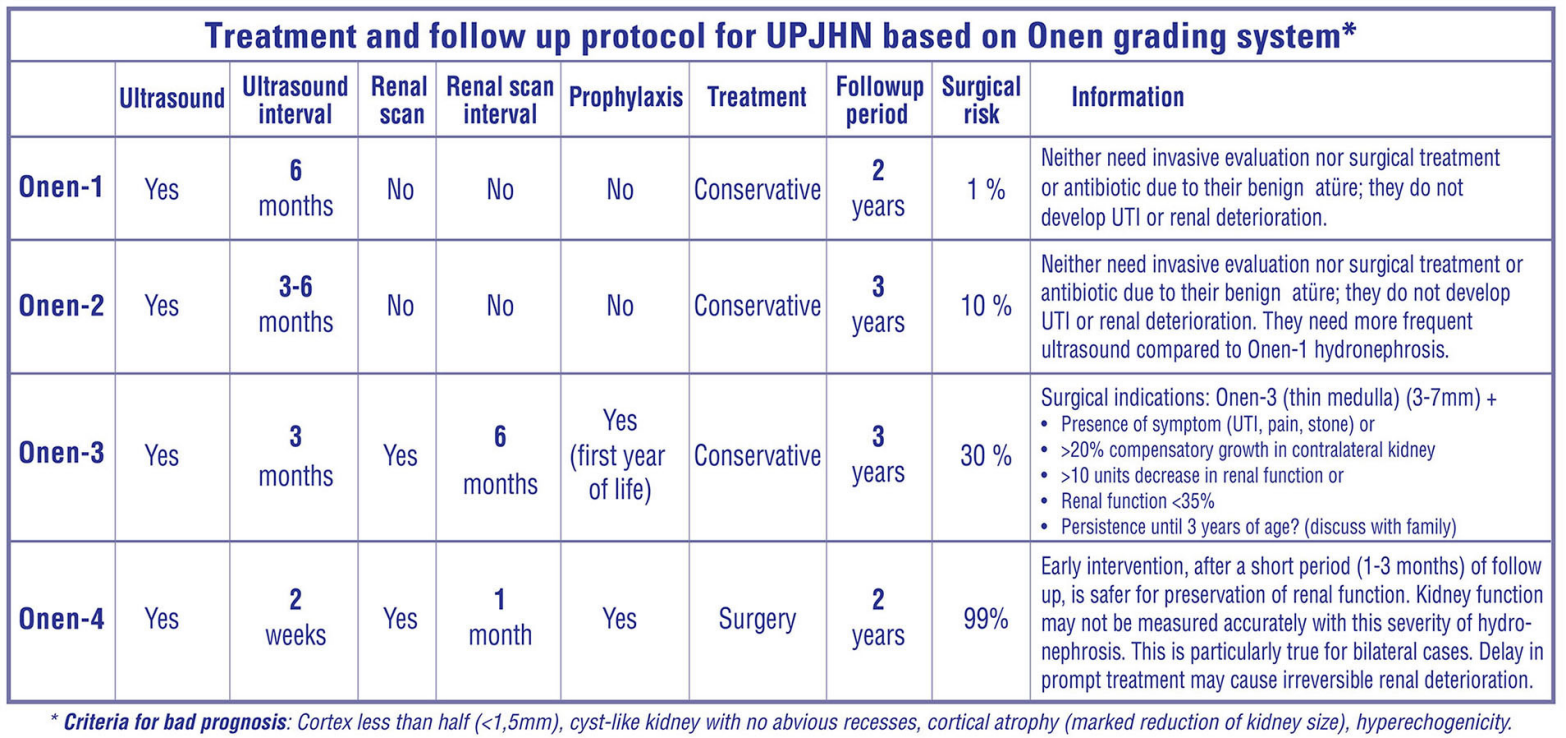
Treatment and follow-up protocol for UPJHN based on the Onen grading system.

- *Onen-1 UPJHN* cases neither need invasive evaluation nor need surgical treatment or antibiotic due to their benign nature; all they need is follow-up with ultrasound alone (Figure 5). A detailed urinary ultrasound at post-natal 1–3–6th months, 1 year, and 2 years of age is enough. If the Onen-1 does not increase or resolve, the follow-up can be ceased.
- *Onen-2 UPJHN* cases neither need invasive evaluation nor need antibiotic due to their benign nature; all they need is follow-up with ultrasound alone. However, about 10% of such infants will worsen and need pyeloplasty during follow-up. Therefore, they might be followed with ultrasound more closely comparing those of Onen-1 hydronephrosis. A detailed urinary ultrasound at post-natal 1–3–6th months and every 6 months until 3 years of age is enough. If Onen-2 decreases to Onen-1 or resolve, the follow-up can be ceased. If Onen-2 persists, an ultrasound might be seen annually until 5 years of age and then the follow-up can be ceased with informing patients about such a symptom as pain or UTI.
- *Onen-3 UPJHN (medulla thin, PK* = *3–7 mm)* patients need close follow-up including renal scan because about one-third of such children need pyeloplasty during follow-up. A detailed urinary ultrasound at post-natal 1st month, every 3 months until 2 years of age, and every 6 months until 3 years of age is reasonable. If the asymptomatic Onen-3 persists until 3 years of age with normal renal function, one of two ways might be discussed with the family; one is continuing invasive follow-up until adulthood, the other is performing a pyeloplasty with high success and thus preventing long-life invasive follow-up and prophylactic antibiotics (1, 5). If Onen-3 is diagnosed, we perform a renal scan. If the function and appearance (on ultrasound) of the ipsilateral kidney as well as contralateral kidney are normal, we follow them and see another ultrasound in 3 months. If Onen-3 decreases or stabilizes, we see the patient in the next 3 months; however, if Onen-3 gets worse, we perform a second renal scan to see renal function. If the function is under 35 or decrease by > 10 units, we perform pyeloplasty. If Onen-3 is diagnosed with renal function under 35, we look at the pictures of the scan in detail. If we believe that the decrease in renal function is correct and the reason of decrease in function is UPJHN, we decide to do surgery because we do not use the washout curve as a treatment criterion. On the other hand, if there is normal clearance of the pelvis and good washout, we look to ultrasound, renal scan, and sometimes VCUG to see if there is any other reason for the hydronephrosis such as a megaureter and reflux.
- *Onen-4 UPJHN (cortex thin, PK*<*3 mm, no corticomedullary differentiation)* patients need surgical correction after a short period of follow-up (1–3 months). Renal function cannot objectively and accurately be assessed with this severity of hydronephrosis. It is particularly true for bilateral once (1, 4–6, 11, 30). Progressive permanent renal damage is inevitable when surgery is delayed in such cases (1, 4–6, 31). On the other hand, timely prompt surgical correction promises to improve decreased renal function in those severe cases (1, 4–6, 11, 30–32). When we see such a neonate with Onen-4, we perform an ultrasound in 1 week of life and then a second ultrasound with MAG3 1 month later. According to the results of these two tests, we decide to perform surgery or follow them conservatively for another month.

## Surgical Indications for Severe Hydronephrosis Associated With Upj Anomalies Based on Grading Systems

In the literature, a surgical decision for UPJHN has been made based on the increase in hydronephrosis on ultrasound in 70% of cases, increase in hydronephrosis on ultrasound, decrease in renal function on scintigraphy in 15%, decrease in renal function on scintigraphy in 10%, and presence of symptom in 5% of UPJHN cases (1). Overall, a surgical decision has been made based on ultrasound findings in 85% of such cases. This rate will even increase if the false-positive findings and misleading (hydration, immobilization, catheterization, position, etc.) of renal scan is taken into account and if nobody uses drainage problems as a surgical indication. Therefore, correct determination of hydronephrosis severity is crucial for infants associated with UPJHN.

### Surgical Indications for UPJHN Based on EAU and ESPU 2019 Guideline

Based on EAU and ESPU 2019 Guidelines on pediatric urology, surgical indications for UPJHN are impaired renal function (<40%), significant renal functional decrease (>10%) in control scans, poor drainage after furosemide injection, increased AP diameter, and SFU-III/IV (33). *All of these indications are problematic:*

- Impaired renal function (<40%) or a decrease in renal function of >10% can be a surgical indication with at least presence of Onen-3 or 4 (thin parenchyma) UPJHN. However, in children with calyceal dilation (Onen-2) alone, the reason of impaired function may be that either an etiology other than UPJHN or the impaired function may actually be false positive.
- Poor drainage function after administration of furosemide by itself should never be used as a surgical indication. This is because the drainage is poor even in UPJHN cases with only calyceal dilation (Onen-2).
- An increased AP diameter on ultrasound by itself should never be used as a surgical indication. It is very discussable. What degree is the increase in AP diameter? How many mm or percent is the increase in AP diameter? At what location of renal pelvis is the AP diameter measured?
- SFU-3 represents only calyceal dilation with the normal renal parenchyma which should never be used as a surgical indication by itself.
- SFU-4 represents any degree of thinning in the renal parenchyma. The wide definition of SFU-4 fails to demonstrate accurately the severity of hydronephrosis and thus significantly misleads from prompt treatment. Those with cortical thinning definitely will need surgery while medullary thinning by itself (with normal renal function) does not need surgery.

### Surgical Indications for UPJHN Based on the Hydronephrosis Severity Score (HSS)

It has been developed to determine the predictivity of pyeloplasty based on ultrasound and diuretic renogram findings (34). The crucial problem and disadvantage of HSS is that it relies on diuretic renogram and its curve. As we all know, renal scan is greatly affected from hydration, bladder catheterization, position, immobilization, function of the affected kidney, laterality (bilateral), diuretic timing, and operator experience (35–38).

### Surgical Indications for UPJHN Based on the Pyeloplasty Prediction Score (PPS)

A recent study has suggested a pyeloplasty prediction score (PPS) using three ultrasound parameters to determine who need surgery and who do not in infants with UPJ-like hydronephrosis (39). They recommend a combination of SFU grade (A), transverse AP diameter (B), and the absolute percentage difference of ipsilateral and contralateral renal lengths at baseline (C) to predict a criterion for surgical need. This study suggests that any infant with UPJO-like hydronephrosis with a PPS of 8 or higher is 8 times more likely to undergo pyeloplasty (39). Unfortunately, none of these parameters is ideal to use due to many disadvantages and/or limitations as described in this review in details. We think that when we put problematic parameters together, it is difficult to get a correct beneficial result from them. Moreover, the laterality (normal right and left long length is different), contralateral or bilateral hydronephrosis, ipsilateral atrophy, or contralateral hypertrophy significantly changes the results of the pyeloplasty prediction score (A + B + C). The absolute percentage (C) would be low when there is a contralateral compensatory growth or an atrophy in ipsilateral kidney which will miss the severity of hydronephrosis. In addition, how would it be an objective criterion in bilateral cases? Any of these parameters can change the percentage (C) from 5 to 20%, which means the score may change from 0 to 4. We should use objective and reproducible criteria that are not affected by many parameters and are applicable for all patients.

### Our Surgical Indications for UPJHN Based on the Onen Grading System

- Onen-4 (thin cortex) (<3 mm)
- Onen-3 (thin medulla) (3–7 mm) plus
- Presence of symptom (UTI, pain, stone) or
- >20% compensatory growth in contralateral kidney or
- >10 units decrease in renal function or
- Renal function <35%.

## Discussion

Although there are many studies in the literature, indications for invasive diagnostics, and surgery in infants with asymptomatic primary UPJHN are an ongoing challenge, and there is no consensus between different disciplines (1, 40). The surgical decision of such patients is done mostly based on ultrasound findings in the literature due to the invasiveness and high negative predictivity of renal scans in infants.

**Figure 6:**
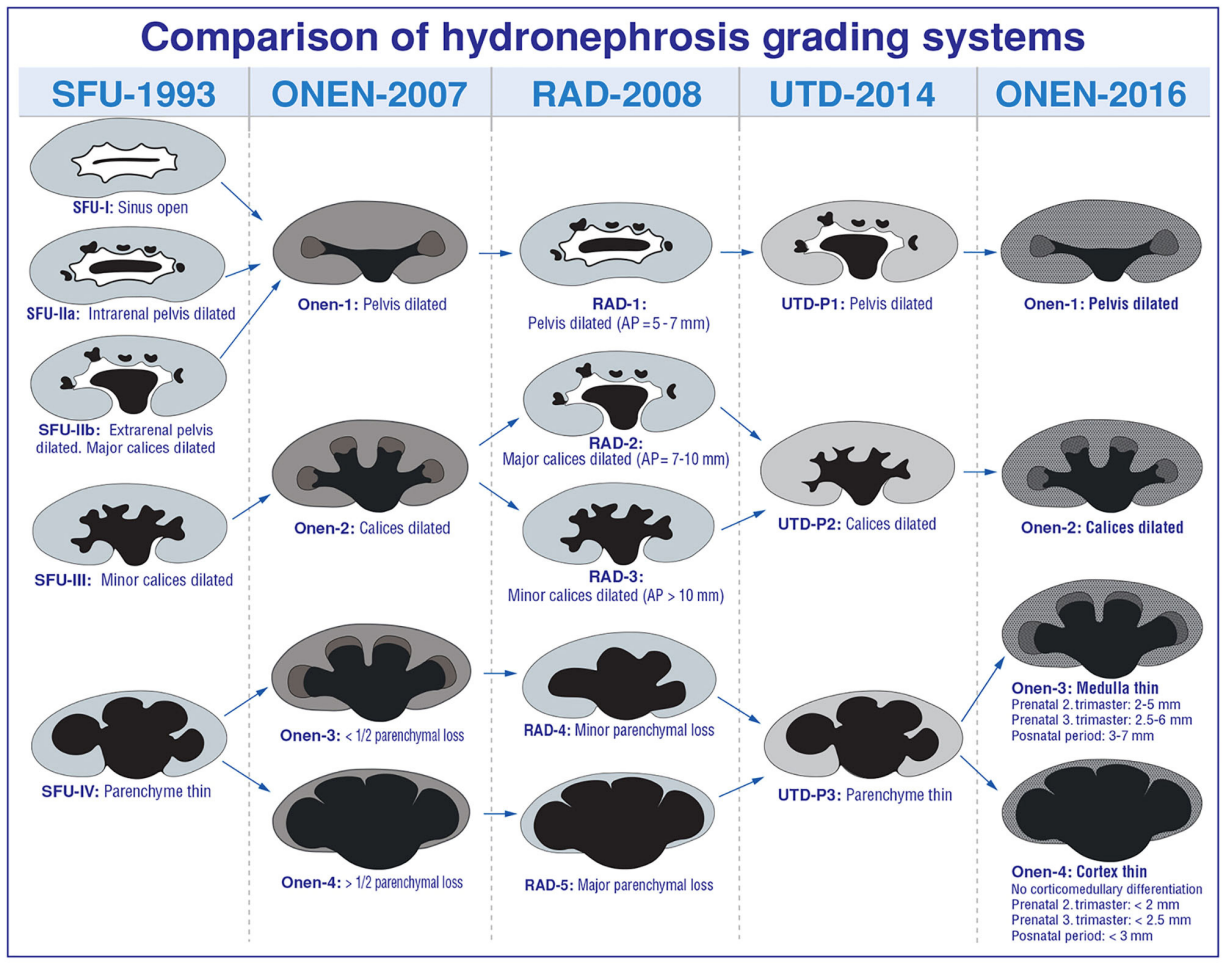
Comparison of hydronephrosis grading systems.

The crucial point for prompt diagnostics, ideal therapeutic approach, and follow-up of such patients is the severity of hydronephrosis. Such many hydronephrosis grading systems as AP diameter, SFU, radiology, UTD, and Onen have been developed to evaluate hydronephrosis severity in infants (1, 2, 4, 7, 9, 18, 23, 40–42) (Figure 6).

Though some authors have proposed cutoff values for the anterior posterior diameter of the renal pelvis, a simple threshold AP diameter value which separates non-obstructive dilation from obstructive dilatation of kidney does not exist (43). AP diameter is a very dynamic parameter and is affected by many factors (1, 10–12, 18, 19). Its measurement is very variable and misleading due to different renal pelvic configurations (1, 4, 10). Therefore, the use of AP diameters has certain disadvantages and limitations. It does not promptly demonstrate the degree of hydronephrosis (1, 2, 4–6, 11, 43). In the literature, there is no study determining intraobserver and interobserver reproducibility of the measurement of AP diameter. In addition, AP diameter does not consider calyceal dilation or the quality of the parenchyma, which may suggest severe cases of obstruction (1, 4, 12, 43).

The radiology grading system has the same grades 1, 2, and 3 as the SFU grading system with addition of the AP diameter for these 3 groups (7, 9). As we discussed above in detail, the AP diameter should not be a parameter in determining the severity of hydronephrosis for many significant reasons. This grading system divides parenchymal loss into two different grades, suggesting the importance of the renal parenchyma to determine the severity of hydronephrosis, which has somewhat a similar idea as that of the Onen hydronephrosis grading system (1, 4, 7). Radiology grade-4 hydronephrosis represents mild parenchymal loss while grade-5 represents severe parenchymal loss (7). However, it is operator dependent, is not decisive, and does not differentiate grades 4 and 5 clearly. Therefore, between- and intra-rater reliability is low.

The SFU grading system has many certain disadvantages and limitations. All grades are problematic and subjective. Both SFU-1 and SFU-2a represent different degrees of renal pelvic dilation. Therefore, it is confusing and is very difficult to differentiate each other (1, 2, 4). Moreover, follow-up, treatment, and prognosis of these two degree are similar; all of them resolve spontaneously without renal damage (1, 2, 4, 11, 20). They should be in the same degree of hydronephrosis. Both SFU-2b and SFU-3 represent different degrees of calyceal dilation (major vs. minor). Therefore, it is subjective, confusing, and very difficult to differentiate each other (1, 4). It can be influenced by the examiner (22). It has modest inter-rater reliability. Although SFU-3 represents only calyceal dilation, the pictures for SFU-3 show severe medullary thinning clearly. This causes significant confusion among the clinicians and radiologists (1, 4). High grades of SFU represent various features, making prognosis difficult to predict (1, 4, 8, 11, 28). It is subjective and can be influenced by the examiner (22). It has modest inter-rater reliability (43).

UTD-P classification appears to be modified partially from SFU and Onen grading system (4, 9). UTD-P1 and 2 have been modified from the Onen grading system (Onen-1 and 2) (4) while UTD-P3 has been modified from the SFU grading system (SFU-4) (9). This classification includes 3 different risk groups: low-risk (UTD-P1), intermediate-risk (UTD-P2), and high-risk (UTD-P3) groups (2, 29). None of these risk groups is the gold standard for all patients. AP diameter >15 mm, peripheral calyceal dilation, and dilated ureter represent intermediate risk (UTD-P2). For example, bilateral grade-4 intra-renal reflux has exactly these findings. However, we all know that this does not represent intermediate risk. It should be in the high-risk group due to the fact that most of these patients develop significant renal damage and UTI breakthrough with fever. There are many similar examples suggesting that this risk scoring is not the standard for all such patients. Between- and within-rater reliability is moderate for this classification (26, 27).

Significant variability exists within and between raters in SFU, radiology grading, and UTD classification. This is because it is significantly operator dependent to differentiate the dilation of peripheral (minor) calices from those of central (major) calices due to a high discrepancy between raters for interpretation of the two types of calyceal dilation (26, 27). Therefore, the UTD score reliability has been found to be low (26, 27). It is exactly the same for SFU-2b and SFU-3 as well as radiology grades 2 and 3 (1, 4, 7, 9, 26, 27). Central (major) calices are somewhat like a neck between the renal pelvis and peripheral (minor) calices. In fact, the real calices are peripheral ones. Therefore, in our opinion, the exact calyceal dilation should be accepted as peripheral (minor) calyceal dilation. It is because it is significant dilation that is clearly different from renal pelvic dilation and is well-visualized and there is no high discrepancy between raters for interpretation (1, 4). Opposite to SFU, radiology, and UTD classification, the Onen grading system does not differ the central and peripheral calyceal dilation.

SFU-4 and UTD-P3 represent the same degree of hydronephrosis. Both represent any kind of renal parenchymal thinning (medulla or cortex), which is a considerable range of severity of hydronephrosis (2, 9). This wide definition of SFU-4 and UTD-P3 fails to demonstrate accurately the severity of hydronephrosis and thus significantly misleads from prompt treatment. They do not suggest who need surgical treatment and who can safely be followed non-operatively in infants with severe UPJHN (1, 2, 4–6, 11). In addition, these two grades make prognosis difficult to predict in UPJHN cases (1, 4, 8, 28).

The Onen hydronephrosis grading system which has been updated in 2016 determined specific detailed findings of significant renal damage, which clearly showed and suggested who can safely be treated conservatively from who will need surgical intervention for UPJHN (1). The intra-rater reliability of Onen grading is higher than that of SFU (2, 20). This grading system has been shown to have good inter- and intra-observer agreements in the diagnosis and follow-up of hydronephrosis in children (20). Intra-observer agreement for the diagnosis of hydronephrosis in prenatal ultrasound recently showed an almost perfect agreement in the Onen grading system (22).

Onen grading system has a sensitivity of 100%, specificity of 76%, and accuracy of 86.4% (21). In a recent study, all units that had Onen-1 and 2 were not obstructed and had renal function > 40% while Onen grade-4 had 100% specificity, meaning that it consistently predicts kidney damage due to obstruction when present (1, 21). Therefore, renal scan is required for only Onen-3 patients; thus, renal scan could be avoided in more than two-thirds of cases (1, 21).

The upgraded Onen grading system not only uses the quality of the renal parenchyma but also takes into account both affected and contralateral kidney size including longitudinal length and atrophy (1). Considering parenchymal loss, SFU and UTD are the same, differing from the Onen grading system that stratifies it in cortical and medullary loss, which was found clearly more precise (1, 21). Recent studies have shown that patients with Onen-3 had better renal function than Onen-4, proving that this difference is relevant to choosing this grading system for children (1, 4, 5, 11, 21). Bienias and Sikora have shown that 21/25 (84%) children with Onen grades 3 and 4 developed obstructive nephropathy with impaired relative function from 15 to 35% (44). If the study separated Onen-3 and 4, almost 100% of Onen-4 would have shown significant renal damage when they did not undergo surgery. Patients with Onen grade-4 had a 100% specificity while those with parenchymal loss not specified (SFU-4, UTD-3) had only 76% specificity regarding obstruction (21). Therefore, dividing SFU-4 or UTD-P3 into Onen grade-3 (medulla thin) and Onen-4 (cortex thin) provides valuable important information in the follow-up and prognosis of high-grade hydronephrosis (1, 4, 8, 11, 21, 28).

DRF and SFU grade of hydronephrosis do not correctly reflect renal injury in bilateral UPJO; however, Onen hydronephrosis grade shows a significant relationship with renal histopathologic grade and can be an indicator for renal injury in UPJO (45). The Onen grading system is more relevant to post-natal prognosis of fetal hydronephrosis compared to SFU and UTD classification (1, 4, 5, 11). It has previously been shown that the Onen grading system determines the severity of UPJHN better and make follow-up more practical compared to SFU and UTD (1, 11). It is reliable and easily reproducible and plays a significant role in the diagnosis of obstruction in children (1, 21). Therefore, the use and popularity of this grading system are increasing around the world (20–22, 45).

In summary, neither AP diameter nor radiology or SFU or UTD is the gold standard in determining the severity of hydronephrosis. They have been shown to be unsuitable for standardizing due to evaluation criteria (1, 4, 21). All these grading systems are based on subjective parameters and are affected by many factors (1, 2, 4–7, 11, 25). They do not determine the exact severity of UPJHN and thus cause permanent renal damage due to delay in surgical decision in some infants while causing unnecessary surgery in others. In addition, they make prognosis difficult in UPJHN cases (1, 4, 8, 11, 28).

The 4 special structures of the kidney (pelvis, calices, medulla, cortex), each having different anatomophysiologic properties, should be taken into account in determining the severity of hydronephrosis. This is because each produces different risks of renal damage. The upgraded Onen hydronephrosis grading system has been developed based on this basic evidence. Therefore, it has resolved all disadvantages of other grading systems. It is an accurate and easily reproducible grading that has high sensitivity and specificity for diagnosis of obstruction, follow-up, prompt treatment (surgical requirement), and prognosis of infants with UPJHN (1, 4–6, 11, 21).

Regardless of the type of hydronephrosis grading systems, AP diameter and calyceal dilation by themselves are insufficient parameters in determining the severity of hydronephrosis. The quality of the renal parenchyma (thickness and appearance) which is the crucial parameter that parallels with renal function and damage should be taken into account in determining the severity of hydronephrosis. This is because it is an important parameter that significantly and objectively suggests who need invasive diagnostic and surgery while giving information about the clinical prognosis of infants associated with UPJHN.

## Author Contributions

The author confirms being the sole contributor of this work and has approved it for publication.

## Conflict of Interest

The author declares that the research was conducted in the absence of any commercial or financial relationships that could be construed as a potential conflict of interest.
